# Association between El Niño-Southern Oscillation events and stroke: a case-crossover study in Kaunas city, Lithuania, 2000–2015

**DOI:** 10.1007/s00484-021-02235-5

**Published:** 2022-01-30

**Authors:** Vidmantas Vaičiulis, Jonė Venclovienė, Giedrė Kačienė, Abdonas Tamošiūnas, Deividas Kiznys, Dalia Lukšienė, Ričardas Radišauskas

**Affiliations:** 1 Department of Environmental and Occupational Medicine, Lithuanian University of Health Sciences, Tilžės St. 18, 47181 Kaunas, Lithuania; 2 Health Research Institute, Lithuanian University of Health Sciences, Tilžės St. 18, 47181 Kaunas, Lithuania; 3 Department of Environmental Sciences, Vytautas Magnus University, Donelaičio St. 58, 44248 Kaunas, Lithuania; 4 Institute of Cardiology, Laboratory of Clinical Cardiology, Lithuanian University of Health Sciences, Sukileliu St. 15, 50103 Kaunas, Lithuania; 5 Institute of Cardiology, Laboratory of Population Studies, Lithuanian University of Health Sciences, Sukileliu St. 15, 50103 Kaunas, Lithuania; 6 Department of Preventive Medicine, Lithuanian University of Health Sciences, Tilžės St. 18, 47181 Kaunas, Lithuania

**Keywords:** ENSO, La Niña, El Niño, Hemorrhagic stroke, Ischemic stroke

## Abstract

The aim of this study was to determine the association between the daily number of cases of ischemic stroke (IS) and hemorrhagic stroke (HS) in patients aged 25–64 years and the El Niño-Southern Oscillation (ENSO) events during 2000–2015. As an indicator of the effect of the ENSO, the monthly NIÑO 3.4 index (Equatorial Pacific Sea Surface Temperature) was used. During the 5844-day study period, 5600 cases of stroke (3170 (56.61%) in men and 2430 (43.39%) in women) were analyzed. Of these, 4354 (77.8%) cases were IS, and 1041 (18.6%) cases were HS. In 3496 (62.2%) cases, stroke occurred in the age group of 55–64 years. In the analysis, we used the following categories of the ENSO events: strong La Niña, moderate La Niña, moderate El Niño, and strong El Niño. The effect of the ENSO was examined by using the multivariate Poisson regression adjusting for weather variables. The highest risk of both strokes (BS) was observed on days of strong and moderate La Niña (rate ratio (*RR*) 1.27, 95% *CI* 1.13–1.42) and *RR* = 1.15 (1.07–1.23), respectively), while the risk for IS was the highest on days of moderate El Niño (*RR* = 1.11(1.02–1.20)). A lower risk for BS was found on days of strong El Niño (*RR* = 0.77(0.62–0.97)). We found that ENSO events affected the occurrence of BS and IS in all age groups, and the strongest effect was observed among females. The results of this study provide new evidence that ENSO events may affect the risk of stroke, especially the risk of IS.

**Supplementary Information:**

The online version contains supplementary material available at 10.1007/s00484-021-02235-5.

## Introduction

There are major differences in stroke incidence rates across Europe. Some of the highest rates are observed in Eastern and Northern Europe (Croatia, Estonia, Lithuania, and Sweden), and some of the lowest are seen in Western and Southern European countries (France, Italy, and Spain) (Stevens et al. 2020).

The risk factors for stroke are well studied, and they include high blood pressure, smoking, obesity, high cholesterol levels, diabetes mellitus, and atrial fibrillation (Feigin et al. 2005; Donnan et al. 2008). However, some literature suggests seasonal variations in the incidence of stroke (Wang et al. 2016), and ambient temperature and relative humidity were identified as risk factors for ischemic stroke (IS) (Mostofsky et al. 2014; Lavados et al. 2018). Magalhães et al. (2011) concluded that even the severity of stroke was associated with weather conditions. Many reports have shown that stroke incidence rates were higher during winter than during the other seasons (Wang et al. 2016; Walach et al. 2002; Fischer et al. 2005), while other studies did not show any significant relationship (Wang et al. 2016; Mostofsky et al. 2014). In other words, the association between the incidence of stroke and meteorological parameters remains controversial.

A variation in ambient temperature, especially heat, can increase the risk of stroke through thromboembolism resulting from hemoconcentration and hyperviscosity, which, in turn, are a consequence of water loss and dehydration (Liu et al. 2015; Lavados et al. 2018). In addition, some studies have shown that cold weather might induce a prothrombotic state within a matter of hours (Nagelkirk et al. 2012). A variation in atmospheric pressure may influence vessel walls and their endothelial function disorders through endogenous inflammatory mechanisms (Jimenez-Conde et al. 2008).

Despite the surge of climate-health-related research in the past decade (Watts et al. 2018), few studies have examined the association between stroke and large-scale climate patterns arising from natural climate variability (Majeed et al. 2021). One of the most important sources of climate variability worldwide is the El Niño Southern Oscillation (ENSO), a coupled atmospheric-ocean phenomenon in the tropical Pacific region with two dominant phases—El Niño (warm) and La Niña (cold), which are well documented in previously published literature (Calvo et al. 2008; Scaife 2010; Timmermann et al. 2018; Dewitte and Takahashi, 2019; Weinberger et al. 2019; Cherchi et al. 2021). The strongest response of the atmospheric pressure and other meteorological parameters near the surface in the Northern Hemisphere (NH) are observed in winter and early spring seasons (Bronnimann 2007). In the Euro-Atlantic region, the El Niño signal usually manifests as an atmospheric pressure dipole, very similar to the negative phase of the North Atlantic Oscillation (NAO) (Brönnimann 2007; Scaife 2010; Mezzina et al. 2020) with a positive anomaly over the pole and Greenland and a band of negative anomalies in mid-latitudes. The NAO is the leading mode of climate variability in the Euro-Atlantic sector, especially in the winter season (Comas-Bru and McDermott 2014; Jakobson et al. 2017; Mellado-Cano et al. 2019). Meteorological parameters near the surface are also determined by other teleconnection patterns, such as the Arctic oscillation, the East Atlantic oscillation, the Scandinavian pattern, and the East Atlantic/West Russia pattern (Bueh and Nakamura 2007; Liu et al. 2014; Ionita 2014; Mikhailova and Yurovsky 2016).

Atmospheric pressure anomalies are accompanied by low-temperature anomalies in Northern Eurasia, La Niña usually showing opposite signals to El Niño (Bronnimann 2007; Scaife, 2010; Weinberger et al. 2019). The associations between the ENSO and precipitation pattern in Europe are much more variable and obscure. Shaman (2014) found a significantly reduced precipitation in summer in the Baltic region and an increased precipitation in the British Isles and Iberia in autumn during El Niño events; however, contradictory observations were shown by other authors (Bronnimann 2007; Scaife, 2010). Previous evidence shows that during the second half of autumn, the NIÑO 3.4 > 1.14 period was characterized by a lower mean atmospheric pressure, more precipitation and relative humidity, and a very significantly lower diurnal temperature range. In winter, the NIÑO 3.4 > 1.14 period was characterized by lower ambient temperature, wind speed, and cloud cover and a higher atmospheric pressure and diurnal temperature range (Venclovienė et al. 2021).

The aim of this study was to detect the complex association between the daily numbers of cases of ischemic (IS) and hemorrhagic (HS) stroke in patients aged 25–64 years. We hypothesized that different ENSO phases have significant and diverse effects on the risk of IS and HS due to the shift in weather conditions such as air temperature, atmospheric pressure, wind speed, and Euro-Atlantic teleconnection patterns.

## Methods

### Stroke events

During the study period (2000–2015), we analyzed data on Kaunas city residents aged 25–64 years. The data were obtained from Kaunas population-based Stroke Registry database. Multiple records from healthcare institutions and different sources of information were evaluated and transferred to form the Stroke Register where stroke events were registered in accordance with the WHO MONICA project protocol and the established quality control procedures (WHO MONICA, 1990). Stroke was defined according to the WHO MONICA protocol (WHO MONICA, 1990). A hemorrhagic stroke (HS) involves the rupture of an arterial vessel, whereas an ischemic stroke (IS) commonly occurs due to reduced cerebral blood flow from obstruction by a dislodged thrombus or atherosclerosis (Grysiewicz et al. 2008). During the study period, stroke types were coded according to ICD-10 classifications: (I61-hemorrhagic stroke (HS), I63-ischemic stroke (IS)). All persons suspected of or having had a non-fatal acute stroke or death from stroke were registered. Regarding the study protocol, every stroke event (HS, IS) had to have its apparent onset within the study period and had to occur more than 28 days from any previously recorded stroke event in the same case. Several stroke attacks occurring within 28 days from the onset were regarded as a single event. Special diagnostic procedures were used for the confirmation of codes for specific types of stroke (HS or IS) (WHO MONICA, 1990). The diagnosis of HS had to be confirmed either by computed tomography (CT) or by autopsy. IS was diagnosed when CT and/or autopsy could verify the infarction and/or exclude hemorrhage and non-vascular disease.

### Data

To indicate the ENSO events (La Nina and El Nino), we used the daily NIÑO 3.4 index interpolated from weekly data (Equatorial Pacific Sea Surface Temperature) from the Climate Explorer database (http://climexp.knmi.nl/data/inino34_weekly.dat). Other weather variables having an effect on stroke occurrence (Vencloviene et al. 2021) were obtained from databases ftp://ftp.cpc.ncep.noaa.gov/cwlinks/ (the daily North Atlantic Oscillation and Arctic Oscillation Indices) and https://www.cpc.ncep.noaa.gov/data/teledoc/telecontents.shtml (the monthly East Atlantic/West Russia pattern and Scandinavian pattern indices). Data on the ambient temperature (°C), atmospheric pressure (hPa), relative humidity (%), and wind speed (m/s) were obtained from Kaunas Meteorological Station.

### Statistical analysis

To compare the mean value of environmental variables, ANOVA was used. Multiple comparisons of the mean values were performed by applying the Scheffe test. As the daily numbers of strokes *Y*_*t*_ are non-negative integers, and their mean values are < 5, we presumed that *Y*_*t*_ followed a Poisson distribution with mean *λ*_*t*_, depending on predictor variables. In the analysis, we used the following categories of the ENSO events: strong La Niña, if Niño 3.4 ≤  − 1.5, moderate La Niña, if − 1.5 < Niño 3.4 ≤  − 0.5, moderate El Niño, if 0.5 ≤ Niño 3.4 < 2, and strong El Niño, if Niño 3.4 ≥ 2. We compared the effect of these ENSO categories with neutral ENSO (− 0.5 < Niño 3.4 < 0.5).

The associations between the ENSO categories and the risk of stroke occurrence were evaluated by using the multivariate Poisson regression model, which was specified as:

$$\mathrm{ln}\left({\lambda }_{t}\right)={\beta }_{0}+{{\varvec{\beta}}}_{1}{{\varvec{X}}}_{t}^{(1)}+{{\varvec{\beta}}}_{2}{{\varvec{X}}}_{t}^{(2)}$$

where *X*^(1)^ was the vector of predictors such as categorical variables (months and week days), the linear trend, population volume, and weather variables set out in our previous work (Vencloviene et al. 2021). For all stroke types, the East Atlantic/West Russia pattern index and a change in daily atmospheric pressure of over 8 hPa from the previous day were additionally included, and for the IS model, a strongly negative East Atlantic/West Russia pattern was additionally included (Breiman et al. 1984). The vector *X*^(2)^ = (*X*^(21)^,…, *X*^(24)^) reflects the ENSO categories such as a strong El Niño, a moderate El Niño, a moderate La Niña, and a strong La Niña, respectively. The reference category is the neutral ENSO. The ***β***_2_ = (*β*_21_, *β*_22_, *β*_23,_ *β*_24_) is a vector of a corresponding regression coefficients. In Poisson regression, the exp(*β*_2i_) is defined as adjusted (for the remaining predictors) rate ratio (*RR*), *i* = 1, 2,…,4.

Rate ratios (RRs) with 95% confidence intervals (95% *CI*) and *p*-values for the association of daily stroke occurrence with the ENSO events were calculated. Statistical analysis was performed using SPSS 20 software.

## Results

During the 16-years study period, 5600 cases of stroke (3170 (56.6%) in men and 2430 (43.4%) in women) were analyzed. Of these, 4354 (77.8%) cases were ischemic stroke (IS) and 1041 (18.6%) cases were hemorrhagic stroke (HS). The majority (3496 (62.2%)) of strokes occurred in the age group of 55–64 years.

According to the data of the NIÑO 3.4 index, a strong La Niña was observed on 332 (5.7%) days, a moderate La Niña—on 1263 (21.6%) days, a moderate El Niño—on 1267 (21.7%) days, and a strong El Niño—on 141 (2.4%) days. The majority (97.3% days) of strong La Niña events occurred during the autumn–winter period, and all of the strong El Niño events were observed during August–December.

**Fig. 1:**
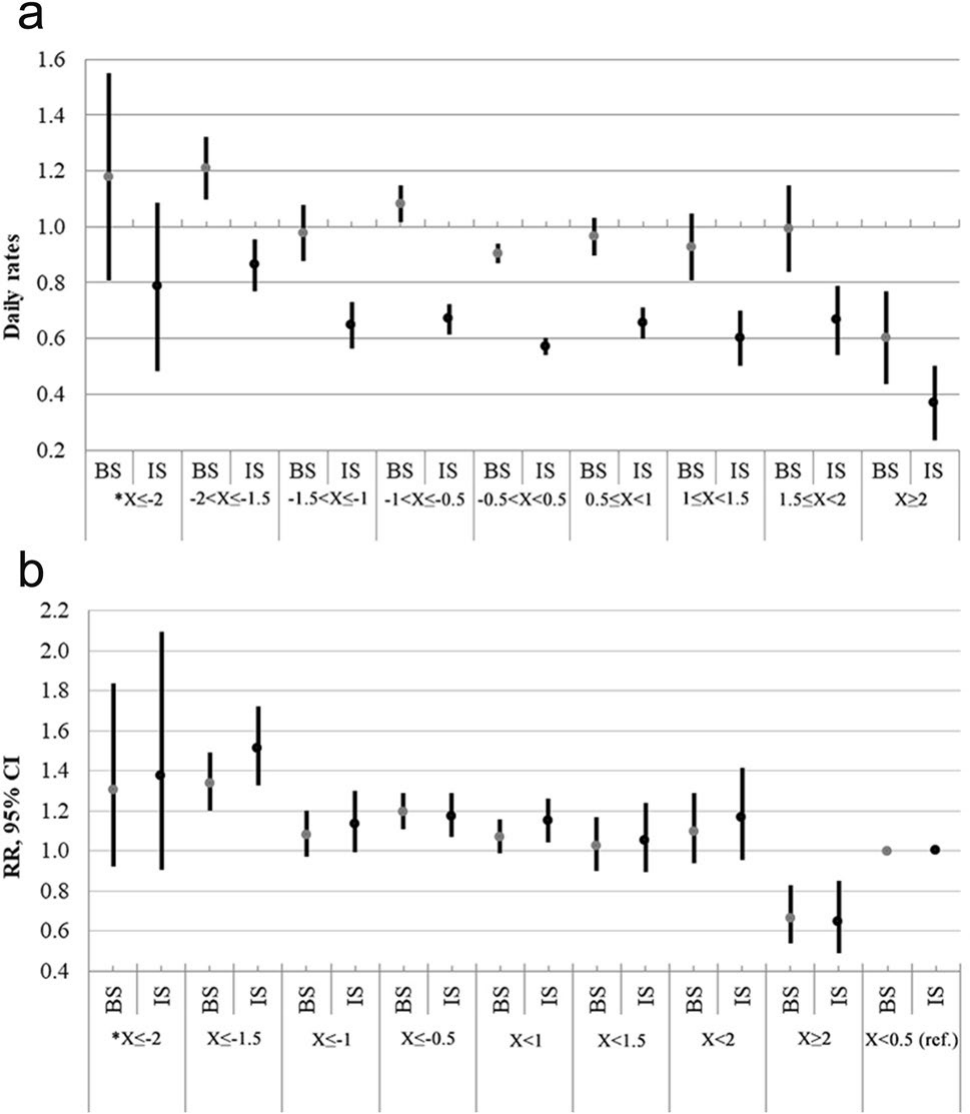
**a** Frequency of both strokes (BS) and ischemic stroke (IS) in the age group of 25–64-year-old adults during different ENSO events in 2000–2015. *X = Niño 3. **b**Associations of different ENSO events with both strokes (BS) and ischemic stroke (IS) in the age group of 25–64-year-old adults in 2000–2015. *X = Niño 3.4

A higher incidence of both strokes (BS) and IS was observed on days of La Niña, and a lower incidence was observed on days of a strong El Niño, whereas during a moderate El Niño, the incidence of BS and IS was higher as compared to that observed during the neutral ENSO (Fig. 1a). The crude rate ratio (RRs) for stroke occurrence was statistically significantly lower on days of a strong El Niño (Niño 3.4 > 2) and on days of La Niña (NIÑO 3.4 ≤  − 0.5) (Fig. 1b).

The results of the analysis of the different groups by sex and age are presented in Tables 1, 2, and 3. We found significant associations between the risk of stroke occurrence and the ENSO events in the age group of 25–64-year-old adults. La Niña events were associated with an increased risk of both stroke types; the association seemed to be stronger during a strong La Niña (*RR* = 1.27, 95% *CI* 1.13–1.42) and during a moderate La Niña (*RR* = 1.15, 95% 1.07–1.23), whereas the risk of BS during strong El Niño events was decreased (*RR* = 0.77, 95% 0.62–0.97). The effect of La Niña and El Niño on the risk of IS was similar to that on the risk of both stroke types (Table 1).

**Table 1:** Associations between different ENSO events and both types of stroke (BS) and ischemic stroke (IS) in males, females, and both sexes aged 25–64 years in Kaunas city in 2000–2015

Stroke	ENSO category		No. of cases	RR (95% CI)	RR (95% CI)	p
				Crude	Adjusted*	
Both sexes	BS	El Niño (strong)	85	0.67 (0.54–0.83)	0.77 (0.62–0.97)	0.026
		El Niño (moderate)	1218	1.06 (0.99–1.14)	1.05 (0.98–1.13)	0.170
		La Niña (moderate)	1326	1.16 (1.09–1.24)	1.15 (1.07–1.23)	< 0.001
		La Niña (strong)	401	1.34 (1.20–1.48)	1.27 (1.13–1.42)	< 0.001
		ENSO (neutral)	2570	**1 [ref.]**		
	IS	El Niño (strong)	53	0.65 (0.49–0.85)	0.67 (0.51–0.89)	0.006
		El Niño (moderate)	939	1.13 (1.04–1.23)	1.11 (1.02–1.20)	0.015
		La Niña (moderate)	1064	1.16 (1.07–1.26)	1.19 (1.10–1.29)	< 0.001
		La Niña (strong)	331	1.50 (1.32–1.70)	1.39 (1.23–1.58)	< 0.001
		ENSO (neutral)	1967	1 [ref.]		
Males	BS	El Niño (strong)	56	0.76 (0.58–0.99)	0.80 (0.61–1.06)	0.127
		El Niño (moderate)	712	1.07 (0.98–1.17)	1.04 (0.95–1.15)	0.404
		La Niña (moderate)	711	1.08 (0.98–1.18)	1.07 (0.97–1.15)	0.182
		La Niña (strong)	203	1.17 (1.01–1.35)	1.10 (0.94–1.28)	0.254
		ENSO (neutral)	1488	1 [ref.]		
	IS	El Niño (strong)	36	0.72 (0.52–1.01)	0.69 (0.49–0.98)	0.036
		El Niño (moderate)	557	1.14 (1.03–1.28)	1.11 (0.99–1.23)	0.064
		La Niña (moderate)	576	1.09 (0.97–1.21)	1.12 (1.01–1.25)	0.029
		La Niña (strong)	174	1.34 (1.13–1.59)	1.25 (1.05–1.49)	0.011
		ENSO (neutral)	1142	1 [ref.]		
Females	BS	El Niño (strong)	29	0.54 (0.37–0.78)	0.71 (0.48–1.04)	0.077
		El Niño (moderate)	506	1.05 (0.94–1.17)	1.07 (0.95–1.19)	0.272
		La Niña (moderate)	615	1.28 (1.16–1.41)	1.26 (1.13–1.39)	< 0.001
		La Niña (strong)	198	1.57 (1.35–1.82)	1.51 (1.28–1.77)	< 0.001
		ENSO (neutral)	1082	1 [ref.]		
	IS	El Niño (strong)	17	0.53 (0.33–0.86)	0.61 (0.37–1.00)	0.051
		El Niño (moderate)	382	1.11 (0.97–1.27)	1.11 (0.97–1.26)	0.124
		La Niña (moderate)	488	1.27 (1.12–1.45)	1.28 (1.14–1.44)	< 0.001
		La Niña (strong)	157	1.73 (1.43–2.09)	1.59 (1.32–1.91)	< 0.001
		ENSO (neutral)	825	1 [ref.]		

^*^RRadjusted for the month, the day of the week, the linear trend, population volume, ambient temperature, and weather/teleconnection variables: the presence of Δ*AT* > 2.2 °C, *RH* on the previous day > 53.5%, *SCAI* > 0.255 for both strokes and *IS*, *EAWR*, and Δ*AP* > 8 hPa for both strokes, and *EAWRI* <  − 1.81 for IS

**Table 2:** Associations between different ENSO events and both types of stroke (BS) and ischemic stroke (IS) in males, females, and both sexes aged 25–54 years in Kaunas city in 2000–2015

Stroke	ENSO category	No. of cases	RR (95% CI)	RR (95% CI)		p
			Crude	Adjusted*		
Both sexes	BS	El Niño (strong)	24	0.51 (0.34–0.77)	0.60 (0.39–0.91)	0.016
		El Niño (moderate)	469	1.11 (1.00–1.24)	1.10 (0.98–1.24)	0.112
		La Niña (moderate)	525	1.25 (1.12–1.39)	1.27 (1.14–1.42)	< 0.001
		La Niña (strong)	140	1.27 (1.06–1.51)	1.26 (1.04–1.53)	< 0.001
		ENSO (neutral)	946	1 [ref.]		
	IS	El Niño (strong)	15	0.53 (0.33–0.86)	0.58 (0.34–0.98)	0.043
		El Niño (moderate)	343	1.11 (0.97–1.27)	1.24 (1.08–1.42)	0.002
		La Niña (moderate)	401	1.27 (1.12–1.45)	1.37 (1.21–1.56)	< 0.001
		La Niña (strong)	108	1.73 (1.43–2.09)	1.42 (1.13–1.77)	0.002
		ENSO (neutral)	664	1 [ref.]		
Males	BS	El Niño (strong)	15	0.56 (0.33–0.93)	0.61 (0.36–1.04)	0.068
		El Niño (moderate)	264	1.09 (0.94–1.26)	1.05 (0.89–1.22)	0.579
		La Niña (moderate)	276	1.14 (0.99–1.32)	1.17 (1.01–1.36)	0.043
		La Niña (strong)	69	1.08 (0.84–1.39)	1.02 (0.78–1.34)	0.868
		ENSO (neutral)	545	1 [ref.]		
	IS	El Niño (strong)	10	0.62 (0.33–1.17)	0.62 (0.32–1.18)	0.147
		El Niño (moderate)	198	1.28 (1.07–1.53)	1.23 (1.02–1.47)	0.027
		La Niña (moderate)	203	1.16 (0.97–1.40)	1.25 (1.05–1.50)	0.012
		La Niña (strong)	55	1.30 (0.96–1.75)	1.25 (0.92–1.70)	0.160
		ENSO (neutral)	377	1 [ref.]		
Females	BS	El Niño (strong)	9	0.45 (0.23–0.88)	0.57 (0.29–1.13)	0.110
		El Niño (moderate)	205	1.15 (0.97–1.36)	1.08 (0.98–1.14)	0.076
		La Niña (moderate)	249	1.40 (1.19–1.64)	1.42 (1.20–1.67)	< 0.001
		La Niña (strong)	71	1.52 (1.18–1.95)	1.61 (1.23–2.12)	0.001
		ENSO (neutral)	401	1 [ref.]		
	IS	El Niño (strong)	5	0.45 (0.19–1.09)	0.50 (0.20–1.24)	0.137
		El Niño (moderate)	145	1.16 (0.93–1.45)	1.24 (1.01–1.53)	0.044
		La Niña (moderate)	198	1.48 (1.20–1.82)	1.52 (1.26–1.84)	< 0.001
		La Niña (strong)	53	1.64 (1.19–2.28)	1.63 (1.18–2.25)	0.003
		ENSO (neutral)	287	1 [ref.]		

^*^RRadjusted for the month, the day of the week, the linear trend, population volume, ambient temperature, and weather/teleconnection variablers: the presence of Δ*AT* > 2.2 °C, *RH* on the previous day > 53.5%, *SCAI* > 0.255 for both strokes and *IS*, *EAWR*, and Δ*AP* > 8 hPa for both strokes, and *EAWRI* <  − 1.81 for IS

**Table 3:** Associations between different ENSO events and both types of stroke (BS) and ischemic stroke (IS) in males, females, and both sexes aged 55–64 years in Kaunas city in 2000–2015

Stroke		ENSO category	No. of cases	RR (95% CI)	RR (95% CI)	p
				Crude	Adjusted*	
Both sexes	BS	El Niño (strong)	61	0.76 (0.59–0.98)	0.98 (0.74–1.32)	0.916
		El Niño (moderate)	749	1.03 (0.95–1.13)	1.05 (0.95–1.15)	0.343
		La Niña (moderate)	801	1.11 (1.02–1.21)	1.04 (0.95–1.14)	0.457
		La Niña (strong)	261	1.38 (1.21–1.57)	1.25 (1.08–1.44)	0.002
		ENSO (neutral)	1624	1 [ref.]		
	IS	El Niño (strong)	38	0.69 (0.50–0.96)	0.82 (0.57–1.18)	0.287
		El Niño (moderate)	596	1.08 (0.97–1.20)	1.05 (0.95–1.17)	0.309
		La Niña (moderate)	663	1.09 (0.99–1.22)	1.06 (0.96–1.17)	0.249
		La Niña (strong)	223	1.53 (1.31–1.78)	1.33 (1.14–1.56)	< 0.001
		ENSO (neutral)	1303	1 [ref.]		
Males	BS	El Niño (strong)	41	0.88 (0.64–1.20)	1.06 (0.74–1.53)	0.745
		El Niño (moderate)	448	1.07 (0.95–1.19)	1.06 (0.94–1.19)	0.337
		La Niña (moderate)	435	1.04 (0.93–1.16)	0.97 (0.85–1.09)	0.581
		La Niña (strong)	134	1.22 (1.02–1.46)	1.10 (0.91–1.34)	0.326
		ENSO (neutral)	943	1 [ref.]		
	IS	El Niño (strong)	26	0.77 (0.52–1.15)	0.88 (0.57–1.37)	0.569
		El Niño (moderate)	359	1.08 (0.94–1.23)	1.06 (0.93–1.21)	0.373
		La Niña (moderate)	373	1.05 (0.91–1.20)	1.01 (0.88–1.15)	0.889
		La Niña (strong)	119	1.37 (1.11–1.68)	1.19 (0.96–1.46)	0.113
		ENSO (neutral)	765	1 [ref.]		
Females	BS	El Niño (strong)	20	0.59 (0.38–0.92)	0.85 (0.52–1.40)	0.517
		El Niño (moderate)	301	0.99 (0.87–1.14)	1.02 (0.89–1.18)	0.746
		La Niña (moderate)	366	1.21 (1.07–1.37)	1.13 (0.98–1.30)	0.087
		La Niña (strong)	127	1.60 (1.32–1.93)	1.45 (1.18–1.79)	< 0.001
		ENSO (neutral)	681	1 [ref.]		
	IS	El Niño (strong)	12	0.57 (0.32–1.01)	0.71 (0.38–1.33)	0.285
		El Niño (moderate)	237	1.08 (0.91–1.28)	1.04 (0.89–1.22)	0.600
		La Niña (moderate)	290	1.17 (0.99–1.37)	1.13 (0.97–1.32)	0.111
		La Niña (strong)	104	1.78 (1.41–2.23)	1.55 (1.23–1.96)	< 0.001
		ENSO (neutral)	538	1 [ref.]		

^*^RR adjusted for the month, the day of the week, the linear trend, population volume, ambient temperature, and weather/teleconnection variables: the presence of Δ*AT* > 2.2 °C, *RH* on the previous day > 53.5%, *SCAI* > 0.255 for both strokes and *IS*, *EAWR*, and Δ*AP* > 8 hPa for both strokes, and *EAWRI* <  − 1.81 for IS

La Niña events were associated only with an increased risk of IS among males (*RR* = 1.25, 95% *CI* 1.05–1.49 during a strong La Niña and *RR* = 1.12, 95% 1.01–1.25 during a moderate La Niña). Moderate El Niño events were also associated with an increased risk of IS in males (*RR* = 1.11, 95% *CI* 0.99–1.23), while a decreased risk of IS occurrence was observed during strong El Niño events (*RR* = 0.69, 95% *CI* 0.49–0.98) (Table 1).

La Niña events were associated with an increased risk of BS in females (*RR* = 1.51, 95% *CI* 1.28–1.77 during a strong La Niña and *RR* = 1.26, 95% *CI* 1.13–1.39 during a moderate La Niña). The effect of La Niña on the risk of IS was similar. A decrease in the risk of IS occurrence was observed during strong El Niño events (*RR* = 0.61, 95% *CI* 0.37–1.00) (Table 1). No associations between ENSO events and the risk of HS were found in females (Table S1) (Supplemental material).

For patients aged 25–54 years (Table 2), the effects of ENSO events were similar to those observed in the age group of 25–64 years. Strong La Niña events were associated with an increased risk of both types of stroke in females aged 25–54 years (*RR* = 1.61, 95% *CI* 1.23–2.12), but not in males. During moderate El Niño events, an increased risk of IS was observed both in females (*RR* = 1.24, 95% *CI* 1.01–1.53) and in males (*RR* = 1.23, 95% *CI* 1.02–1.47) (Table 2). No associations between ENSO events and the risk for HS were found (Table S2) (Supplemental material).

Strong La Niña events were associated with an increased risk of both types of stroke (*RR* = 1.25, 95% *CI* 1.08–1.44) and IS (*RR* = 1.33, 95% 1.14–1.56) in all adults aged 55–64 years. This effect was stronger in females than in males (Table 3). No associations between ENSO events and the risk of HS were found (Table S3) (Supplemental material).

The mean values of weather variables and teleconnection indices as well as the results of the multiple comparison in the ANOVA are presented in Table 4. The period of La Niña events was characterized by lower ambient temperature and East Atlantic/West Russia pattern indices as compared to the neutral or warm ENSO. Lower ambient temperature and a higher relative humidity and wind speed were observed during strong La Niña events as compared to other categories of the ENSO. Lower ambient temperature and Arctic Oscillation Indices and higher relative humidity and East Atlantic/West Russia pattern indices were observed during moderate El Niño events. The periods of a strong El Niño were characterized by higher atmospheric pressure, North Atlantic Oscillation Indices, and Arctic Oscillation Indices and lower wind speed as compared to other groups of ENSO. Apart from this, the mean values of ambient temperature and relative humidity during strong El Niño events were closer to those during the neutral ENSO as compared to moderate El Niño events, but no statistical significance was found (Table 4).

**Table 4:** The distribution of seasons and mean values of weather variables and teleconnection indices during different ENSO events*

Variable	Strong La Niña (1)	Moderate La Niña (2)	Neutral ENSO (3)	Moderate El Niño (4)	Strong El Niño (5)
Winter	208 (62.7)	446 (35.3)	437 (15.4)	322 (25.4)	31 (22.0)
Spring	1 (0.3)	352 (27.9)	938 (33.0)	181 (14.3)	0
Summer	8 (2.4)	221 (17.5)	881 (31.0)	343 (27.1)	19 (13.5)
Autumn	115 (34.6)	244 (19.3)	585 (20.6)	421 (33.2)	91 (64.5)
*Minimal AT, °C	− 1.27 (2,3,4,5)	1.77 (1,3,4,5)	5.27 (1,2,4)	3.92 (1,2,3)	4.98 (1,2)
*Mean AT, °C	1.30 (2,3,4,5)	5.16 (1,3,4,5)	9.50 (1,2,4)	7.61 (1,2,3)	8.53 (1,2)
*Maximal AT, °C	3.93 (2,3,4,5)	8.85 (1,3,4,5)	14.06 (1,2,4)	11.6 (1,2,3)	12.42 (1,2)
*RH, %	87.1 (2,3,4,5)	81.7 (1,3)	77.9 (1,2.4)	81.3 (1,3)	79.7 (1)
*AP, hPa	1015 (5)	1015 (5)	1015 (5)	1016 (5)	1019 (1,2,3,4)
*WS	5.82 (2,3,4,5)	5.23 (1,4,5)	5.08 (1,4,5)	4.79 (1,2,3,5)	2.80 (1,2,3,4)\
*NAOI	− 0.10 (2,5)	0.11 (1,3,4,5)	0.05 (2,5)	− 0.09 (2,5)	0.36 (1,2,3,4)
*AOI	− 0.18 (5)	0.02 (4,5)	0.01 (4,5)	− 0.18 (2,3,4)	0.51 (1,2,3,4)
*EA/WRI	− 0.43 (3,4,5)	− 0.38 (3,4,5)	− 0.24 (1,2,4,5)	0.11 (1,2,3)	0.13 (1,2,3)
*SCAI	− 0.09 (2,5)	0.19 (1,3,4)	− 0.01 (2,5)	0.00 (2,5)	0.42 (1,3,4)

^*^The codes in parentheses indicate the groups of ENSO events whose mean values differ statistically significantly. *AT* ambient temperature, *RH* relative humidity, *AP* atmospheric pressure, *WS* wind speed, *NAOI* North Atlantic Oscillation Indices, *AOI* Arctic Oscillation Indices, *EA/WRI* East Atlantic/West Russia pattern indices, *SCAI* Scandinavian pattern indices

## Discussion

This case-crossover population-based study provided evidence that ENSO events increased the risk of stroke. We analyzed the effect of both El Niño and La Niña defined as categorical variables reflecting moderate and strong events. We compared the effect of these ENSO categories with that of the neutral ENSO (− 0.5 < NIÑO 3.4 < 0.5) and found an increased risk of both investigated strokes and IS during moderate La Niña and strong La Niña events. The effect of El Niño was also different during moderate and strong events; however, these effects acted as contraries, especially for IS: the risk of IS was higher during a moderate El Niño and lower during a strong El Niño. A decreased risk during a strong El Niño was also found for all types of stroke in both males and females. Significant associations between the risk of stroke and ENSO events were observed in all age groups (25–64, 25–54, and 55–64 years). The effect estimates were greater among females than among males in all age groups.

We found statistically significant effects of ENSO events when controlling for the effect of the month, population volume, and statistically significant weather variables (ambient temperature, atmospheric pressure, and relative humidity). These effects may be explained by different weather patterns during the ENSO events in the troposphere and the stratosphere. Even though there are extensive studies investigating the association between ambient temperature and the risk of stroke, the significance of other meteorological risk factors, such as atmospheric pressure and relative humidity, has been less frequently explored. The results from several studies on atmospheric pressure and relative humidity were inconsistent (Cao et al. 2016).

The effect of the ENSO on meteorological conditions in remote areas underlies the effects on human health, and this effect is dependent on the ocean-troposphere coupling near the equator and the transduction of the ENSO signal to higher latitudes. The canonical ENSO signal is transferred by the stratospheric pathway (Bell et al. 2009). The strongest ENSO signal is found in the boreal winter, when stratospheric winds are westerly in NH (Calvo et al. 2008). El Niño conditions usually result in the weakening of the polar vortex and in turn contributes to a sudden stratospheric warming (SSW) and a breakdown of the NH polar vortex in late winter and spring (Bell et al. 2009; Garfinkel and Hartmann 2007; Limpasuvan et al. 2004; Scaife 2010; Weinberger et al. 2019). The weakening in the polar vortex and SSW events initiate the negative North Atlantic Oscillation (NAO) phase and low temperature anomalies in winter and early spring in the North European region, which are associated with weaker than average westerly winds across the middle latitudes (Comas-Bru and McDermott 2014) and cold spells in Europe (Scaife 2010; Tomassini et al. 2012).

The analysis of the mean values of weather variables in Kaunas city during the study period showed that La Niña events were characterized by lower ambient temperature and East Atlantic/West Russia pattern indices, and strong La Niña events lead to lower (higher) temperature, relative humidity, and wind speed as compared to other categories of ENSO. The period of moderate El Niño was also characterized by a lower ambient temperature and a higher relative humidity as compared to the neutral ENSO. Previous evidence from studies conducted in different climate regions such as Central and Western Europe and Asia had shown that a lower ambient temperature increased the risk of stroke or IS in both sexes and different age groups (Matsumaru et al. 2020; Mostofsky et al. 2014; Luo et al. 2018; Wang et al. 2013; Rakers et al. 2016; Ravljen et al. 2021).

Even though the studies are few, their results are quite contradictory and are not always in line with the results of our study. South American scientists performed a systematic review and concluded that whatever the exposure or the outcome, older ages were at an increased risk. Sex did not respond equally to ambient temperature: women were at an increased risk of stroke at colder temperatures, whereas men were more vulnerable at hotter temperatures (Lavados et al. 2018). We also found a higher risk of stroke in females, especially during a strong La Niña in the age group of 55–64 years. During La Niña events, the risk of IS was increased both in males and females; however, the risk of HS was not increased in males and was increased only at 93% of significance in females.

This could be associated with lower ambient temperatures during La Niña, especially the strongest events. There are several mechanisms that may explain the link between colder ambient temperature and IS. Exposure to cold increases vasoconstriction and blood pressure, platelet count, cholesterol, heart rate, plasma fibrinogen, platelet viscosity, and peripheral vasoconstriction (Halonen et al. 2011; Schauble et al. 2012; Hong et al. 2012).

One more study conducted in the USA found that the relationship between a lower ambient temperature and higher stroke rates was similar across the seasons and was stronger on days with a higher relative humidity, suggesting that ambient temperature variables may be linked with the risk of stroke (Cowperthwaite and Burnett 2011). A study conducted in Israel also found a higher risk of IS on days with higher levels of relative humidity (Mostofsky et al. 2014). Meanwhile, two studies in China and one in Slovenia showed no significant association between relative humidity and hospitalizations for IS (Wang et al. 2013; Qi et al. 2020; Ravljen et al. 2021). Therefore, the effect of relative humidity on the risk of stroke is controversial, showing both an increased (Rakers et al. 2016) and a negligible (Cao et al. 2016) risk of stroke.

The influence of atmospheric pressure on the incidence of stroke has been reported in a small number of studies. Most of these studies found statistically significant associations between an increase in atmospheric pressure and the risk of different types of stroke (McArthur et al. 2010). Conversely, Rakers et al. (2016) found a decreased (by 14%) overall risk of stroke in younger individuals. The physiological mechanism for any association between atmospheric pressure and stroke is not clear, and particularly little evidence exists for IS. The exact pathophysiological mechanism for the associations between atmospheric pressure and stroke are still unknown (Lim et al. 2017). However, some studies make assumptions that atmospheric pressure may directly influence vessel walls, triggering endogenous inflammatory mechanisms, and changing their endothelial function (Jimenez-Conde et al. 2008). Furthermore, it is thought that changes in atmospheric pressure may lead to a plaque rupture in the carotid arteries (Houck et al. 2005). Studies on thrombosis in air travel suggest that prothrombin fragments and the thrombin-antithrombin complex are activated in hypobaric conditions (Gungor and Onar 2007; Schreijer et al. 2006), which could be another clue to the underlying mechanism (Vencloviene et al. 2021).

The results obtained in previous studies showed different and controversial effects of atmospheric pressure on HS and IS. It is possible that the divergent effect of the ENSO on the risk IS and HS is due to the different effects of the ENSO on European atmospheric variation, including a shift in atmospheric pressure. The analysis of the means of weather variables in Kaunas city during different ENSO categories did not show any differences in daily changes of atmospheric pressure (*p* > 0.9).

We found a different and significant effect of strong and moderate ENSO on stroke. Our findings provide additional insight into a similar association in Lithuania where some evidence was found on a link between atmospheric circulation and health (Venclovienė et al. 2021). The weather conditions during the period of a strong El Niño were different as compared to the period of a moderate El Niño. During a strong El Niño, a higher (lower) atmospheric pressure, North Atlantic Oscillation Indices, and Arctic Oscillation Indices (wind speed) were observed. Other authors have also observed that strong El Niño events created a higher atmospheric pressure, a lower ambient temperature, and dry air in the Baltic countries (Moron and Guy 2003; Fraedrich 1994; King et al. 2020). It is probable that these weather patterns positively affected the health of the population with a higher risk of stroke.

The different effects of strong and moderate El Niño events on HS and IS incidence rates might be explained by nonlinearity in the intensity of the tropospheric and stratospheric response in NH to moderate and strong El Niño events. Several authors have demonstrated a clear non-linearity of the response of European winter and/or spring atmospheric pressure to the El Niño amplitude (Bell et al. 2009; Scaife 2010; Weinberger et al. 2019), leading to a deviation of surface response from the canonical signal in the case of the strongest El Niño events. Scaife (2010) claimed that only moderate to weak events show the canonical negative NAO pattern, while the strongest events show a rather different pattern with a barotropic high moved to the west of Europe. The results of our study suggest that a shift in meteorological conditions during a strong El Niño, such as markedly lower wind speed, higher atmospheric pressure, and indices of NAO and AO, have a significant reducing effect of the risk of stroke.

The geographical position is a significant confounding variable in the risk of stroke due to acclimatization (Lavados et al. 2018). The controversy between our results and the preceding ones could be related to differences in the analyzed populations and the major comorbidities, but the differences in methodology can also be of importance. In addition, we did not have the opportunity to evaluate patients older than 65 years. There are only a handful of studies on the impact of ambient temperature on HS (Han et al. 2016). A study in South Korea showed that there was no significant correlation between mean ambient temperature and HS, with an exception of a negative correlation between ambient temperature and HS in the older age group (Han et al. 2016).

A limitation of our investigation was that even though the Kaunas population-based Stroke Register database is prospective, it still registers cases after a delay. It is possible that some cases registered in Kaunas city during 2000–2015 were not captured by that method. However, given the wide data collection protocol, this number is likely to be too small to influence our results.

One more limitation of this study is that we did not analyze the importance of air pollution on stroke because air pollutants can be seen as interim variables in the pathway from different weather to stroke (Liu et al. 2021). Adjusting for the interim variables would lead to the underestimation of the real effect. Air pollution levels in Kaunas city are also relatively low, and thus its importance in mediating the effects of ENSO was likely small in this study (The environmental implementation review 2019; Kaunas city environment monitoring 2014).

One more limitation of the study is that we did not adjust for other confounding factors (including daily physical activity, smoking, and other behavioral factors), the effects of other pre-existing diseases (hypertension, diabetes, or a previous stroke), or medication use, which, according to different sources, could theoretically be effect modifiers or intermediates, but not confounders (Majeed et al. 2020; Obradovich and Fowler 2017; Vaiciulis et al. 2021).

This research has some strengths. We applied uniform suitability criteria based on the WHO MONICA criteria, and the order of case selection did not change.

Another strength of this research is the application of the case-crossover design. The case-crossover design is suited for studying transient risk factors over time and space (Maclure 1991), and the present application controls for time-invariant and time-varying confounders. In conclusion, it should be pointed out that the rather short duration of accurate meteorological observations and a relatively low number of exceptionally strong El Niño events limits a more accurate determination of the effects of the ENSO on NH climate—and especially on health. However, this case-crossover study provided evidence that depending on the categories of the ENSO events, the risk of stroke increased. The highest risk of stroke in the age group of 25–64 years was observed during La Niña (strong) events. The associations did not seem equally strong in both IS and HS. Females seemed to be more sensitive to the effects of the ENSO than males were, but there was no difference in the effect by age. More studies are necessary on factors influencing sensitivity to stroke during the ENSO so that targeted interventions could become evidence-based.

## Supplementary Information

Below is the link to the electronic supplementary material.

Supplementary file1 (DOCX 38 KB)

## Data Availability

The data on stroke cases were obtained from the Institute of Cardiology at the Lithuanian University of Health Sciences. Data are not accessible online. Monthly NIÑO 3.4 indices were taken from the Climate Explorer database (https://climexp.knmi.nl/data/iersst_nino3.4a.dat). The values of the daily North Atlantic Oscillation and Arctic Oscillation Indices (North Atlantic Oscillation Indices and Arctic Oscillation Indices) were obtained from the National Oceanic and Atmospheric Administration database ftp://ftp.cpc.ncep.noaa.gov/cwlinks/. The monthly East Atlantic/West Russia pattern and Scandinavian pattern indices were obtained from the database of the National Oceanic and Atmospheric Administration (https://www.cpc.ncep.noaa.gov/data/teledoc/telecontents.shtml).
