# Supplementary material for: Association between El Niño-Southern Oscillation events and stroke: a case-crossover study in Kaunas city, Lithuania, 2000–2015
Source: Int J Biometeorol. 2022 Jan 30;66(4):769–79. doi: 10.1007/s00484-021-02235-5 (PMC8948119; doi:10.1007/s00484-021-02235-5)
Supplement: Supplementary file 1 — Supplementary file1 (DOCX 38 KB) [file 484_2021_2235_MOESM1_ESM.docx]

**Supplemental Material**

**Association between El Niño-Southern Oscillation Events and Stroke: A Case-Crossover Study in Kaunas City, Lithuania, 2000-2015**

Vidmantas Vaičiulis*, Jonė Venclovienė, Giedrė Kacienė Abdonas Tamošiūnas, Deividas Kiznys, Dalia Lukšienė, Ričardas Radišauskas

**Table S1.** Associations between different ENSO events and hemorrhagic stroke in males, females and both sexes aged 25-64 years in Kaunas, 2000-2015

| **Stroke** | **ENSO Category** | **No. of Cases** | **RR (95%CI)** | | **RR (95%CI)** | **p** |
| --- | --- | --- | --- | --- | --- | --- |
|  |  |  | **Crude** | | **Adjusted*** | |
| Both sexes | El Nino (strong) | 20 | 0.75 (0.42-1.33) | | 0.93 (0.58-1.50) | 0.763 |
|  | El Nino (moderate) | 242 | 1.24 (1.03-1.48) | | 0.99 (0.84-1.17) | 0.901 |
|  | La Nina (moderate) | 227 | 1.03 (0.85-1.25) | | 1.05 (0.89-1.23) | 0.601 |
|  | La Nina (strong) | 60 | 1.01 (0.72-1.41) | | 0.91 (0.69-1.21) | 0.518 |
|  | ENSO (neutral) | 492 | **1 [ref.]** | |  |  |
| Males | El Nino (strong) | 10 | 0.82 (0.40-1.66) | | 0.82 (0.42-1.60) | 0.552 |
|  | El Nino (moderate) | 131 | 1.25 (0.99-1.58) | | 0.93 (0.74-1.16) | 0.518 |
|  | La Nina (moderate) | 113 | 0.96 (0.74-1.24) | | 0.90 (0.72-1.13) | 0.363 |
|  | La Nina (strong) | 25 | 0.65 (0.39-1.10) | | 0.67 (0.44-1.02) | 0.064 |
|  | ENSO (neutral) | 280 | **1 [ref.]** | |  |  |
| Females | El Nino (strong) | 10 | 0.64 (0.24-1.73) | 1.08 (0.55-2.14) | | 0.816 |
|  | El Nino (moderate) | 111 | 1.21 (0.90-1.63) | 1.07 (0.84-1.37) | | 0.580 |
|  | La Nina (moderate) | 114 | 1.14 (0.85-1.54) | 1.24 (0.98-1.57) | | 0.073 |
|  | La Nina (strong) | 35 | 1.56 (1.00-2.44) | 1.24 (0.84-1.81) | | 0.281 |
|  | ENSO (neutral) | 212 | **1 [ref.]** |  | |  |

* RR adjusted for the month, the day of the week, the linear trend, population volume, ambient temperature, and weather/teleconnection variables: the presence of ΔAT >2.2 ^o^C, RH on the previous day >53.5%, SCAI >0.255 for both strokes and IS, EAWR, and ΔAP >8 hPa for both strokes, and EAWRI <-1.81 for IS.

**Table S2.** Associations between different ENSO events and hemorrhagic stroke in males, females and both sexes aged 25-54 years in Kaunas, 2000-2015

| **Stroke** | **ENSO Category** | **No. of Cases** | **RR (95%CI)** | **RR (95%CI)** | **p** |
| --- | --- | --- | --- | --- | --- |
|  |  |  | **Crude** | **Adjusted*** | |
| Both sexes | El Nino (strong) | 6 | 0.58 (0.21-1.56) | 0.55 (0.24-1.27) | 0.160 |
|  | El Nino (moderate) | 109 | 1.07 (0.80-1.44) | 0.88 (0.69-1.12) | 0.285 |
|  | La Nina (moderate) | 116 | 1.09 (0.82-1.46) | 1.05 (0.83-1.32) | 0.692 |
|  | La Nina (strong) | 29 | 1.10 (0.67-1.80) | 0.90 (0.60-1.35) | 0.602 |
|  | ENSO (neutral) | 248 | **1 [ref.]** |  |  |
| Males | El Nino (strong) | 3 | 0.66 (0.21-2.08) | 0.46 (0.14-1.50) | 0.197 |
|  | El Nino (moderate) | 55 | 1.02 (0.71-1.48) | 0.71 (0.51-0.99) | 0.045 |
|  | La Nina (moderate) | 67 | 1.05 (0.73-1.51) | 0.98 (0.72-1.32) | 0.873 |
|  | La Nina (strong) | 13 | 0.74 (0.36-1.53) | 0.61 (0.34-1.10) | 0.103 |
|  | ENSO (neutral) | 147 | **1 [ref.]** |  |  |
| Females | El Nino (strong) | 3 | 0.42 (0.06-3.04) | 0.70 (0.21-2.33) | 0.560 |
|  | El Nino (moderate) | 54 | 1.17 (0.72-1.89) | 1.14 (0.80-1.63) | 0.478 |
|  | La Nina (moderate) | 49 | 1.17 (0.72-1.90) | 1.14 (0.80-1.63) | 0.458 |
|  | La Nina (strong) | 16 | 1.78 (0.90-3.52) | 1.42 (0.80-2.53) | 0.235 |
|  | ENSO (neutral) | 101 | **1 [ref.]** |  |  |

* RR adjusted for the month, the day of the week, the linear trend, population volume, ambient temperature, and weather/teleconnection variables: the presence of ΔAT >2.2 ^o^C, RH on the previous day >53.5%, SCAI >0.255 for both strokes and IS, EAWR, and ΔAP >8 hPa for both strokes, and EAWRI <-1.81 for IS.

**Table S3.** Associations between different ENSO events and hemorrhagic stroke in males, females and both sexes aged 55-64 years in Kaunas, 2000-2015

| **Stroke** | **ENSO Category** | **No. of Cases** | **RR (95%CI)** | **RR (95%CI)** | **p** |
| --- | --- | --- | --- | --- | --- |
|  |  |  | **Crude** | **Adjusted*** | |
| Both sexes | El Nino (strong) | 14 | 0.88 (0.43-1.79) | 1.34 (0.70-2.56) | 0.737 |
|  | El Nino (moderate) | 133 | 1.36 (1.07-1.72) | 1.16 (0.93-1.45) | 0.188 |
|  | La Nina (moderate) | 111 | 0.98 (0.76-1.28) | 1.01 (0.79-1.29) | 0.938 |
|  | La Nina (strong) | 31 | 0.94 (0.59-1.48) | 0.98 (0.66-1.46) | 0.920 |
|  | ENSO (neutral) | 244 | **1 [ref.]** |  |  |
| Males | El Nino (strong) | 7 | 0.96 (0.39-2.35) | 1.18 (0.48-2.88) | 0.723 |
|  | El Nino (moderate) | 76 | 1.45 (1.07-1.97) | 1.23 (0.92-1.67) | 0.169 |
|  | La Nina (moderate) | 46 | 0.88 (0.61-1.26) | 0.80 (0.56-1.15) | 0.228 |
|  | La Nina (strong) | 12 | 0.57 (0.27-1.23) | 0.80 (0.43-1.50) | 0.491 |
|  | ENSO (neutral) | 133 | **1 [ref.]** |  |  |
| Females | El Nino (strong) | 7 | 0.78 (0.25-2.46) | 1.61 (0.63-4.10) | 0.318 |
|  | El Nino (moderate) | 57 | 1.24 (0.85-1.79) | 1.08 (0.77-1.52) | 0.640 |
|  | La Nina (moderate) | 65 | 1.13 (0.77-1.65) | 1.24 (0.89-1.73) | 0.202 |
|  | La Nina (strong) | 19 | 1.43 (0.79-2.57) | 1.14 (0.68-1.94) | 0.617 |
|  | ENSO (neutral) | 111 | **1 [ref.]** |  |  |

* RR adjusted for the month, the day of the week, the linear trend, population volume, ambient temperature, and weather/teleconnection variables: the presence of ΔAT >2.2 ^o^C, RH on the previous day >53.5%, SCAI >0.255 for both strokes and IS, EAWR, and ΔAP >8 hPa for both strokes, and EAWRI <-1.81 for IS.
